# Vocal Experimentation in the Juvenile Songbird Requires a Basal Ganglia Circuit

**DOI:** 10.1371/journal.pbio.0030153

**Published:** 2005-03-29

**Authors:** Bence P Ölveczky, Aaron S Andalman, Michale S Fee

**Affiliations:** **1**McGovern Institute for Brain Research, Department of Brain and Cognitive SciencesMassachusetts Institute of Technology, Cambridge, MassachusettsUnited States of America; **2**Harvard Society of Fellows, Harvard UniversityCambridge, MassachusettsUnited States of America; University of CambridgeUnited Kingdom

## Abstract

Songbirds learn their songs by trial-and-error experimentation, producing highly variable vocal output as juveniles. By comparing their own sounds to the song of a tutor, young songbirds gradually converge to a stable song that can be a remarkably good copy of the tutor song. Here we show that vocal variability in the learning songbird is induced by a basal-ganglia-related circuit, the output of which projects to the motor pathway via the lateral magnocellular nucleus of the nidopallium (LMAN). We found that pharmacological inactivation of LMAN dramatically reduced acoustic and sequence variability in the songs of juvenile zebra finches, doing so in a rapid and reversible manner. In addition, recordings from LMAN neurons projecting to the motor pathway revealed highly variable spiking activity across song renditions, showing that LMAN may act as a source of variability. Lastly, pharmacological blockade of synaptic inputs from LMAN to its target premotor area also reduced song variability. Our results establish that, in the juvenile songbird, the exploratory motor behavior required to learn a complex motor sequence is dependent on a dedicated neural circuit homologous to cortico-basal ganglia circuits in mammals.

## Abstract 2

Electrophysiological recording and pharmacological inactivation suggest that variable activity in area LMAN of the zebrafinch contributes to the exploratory motor behaviour required for song learning

## Introduction

The acquisition of complex motor sequences, such as swinging a golf club or playing the piano, can be thought of as reinforcement learning. This learning process requires the exploration of a range of motor actions and the concomitant evaluation of the resulting performance, reinforcing motor programs that lead to improved outcomes [1]. Similarly, juvenile songbirds explore a large range of vocalizations by continuously varying their song [2], utilizing auditory feedback to improve their performance [3]. Thus, song learning encompasses the two ingredients of reinforcement learning: exploratory motor behavior, and performance evaluation.

**Figure 1:**
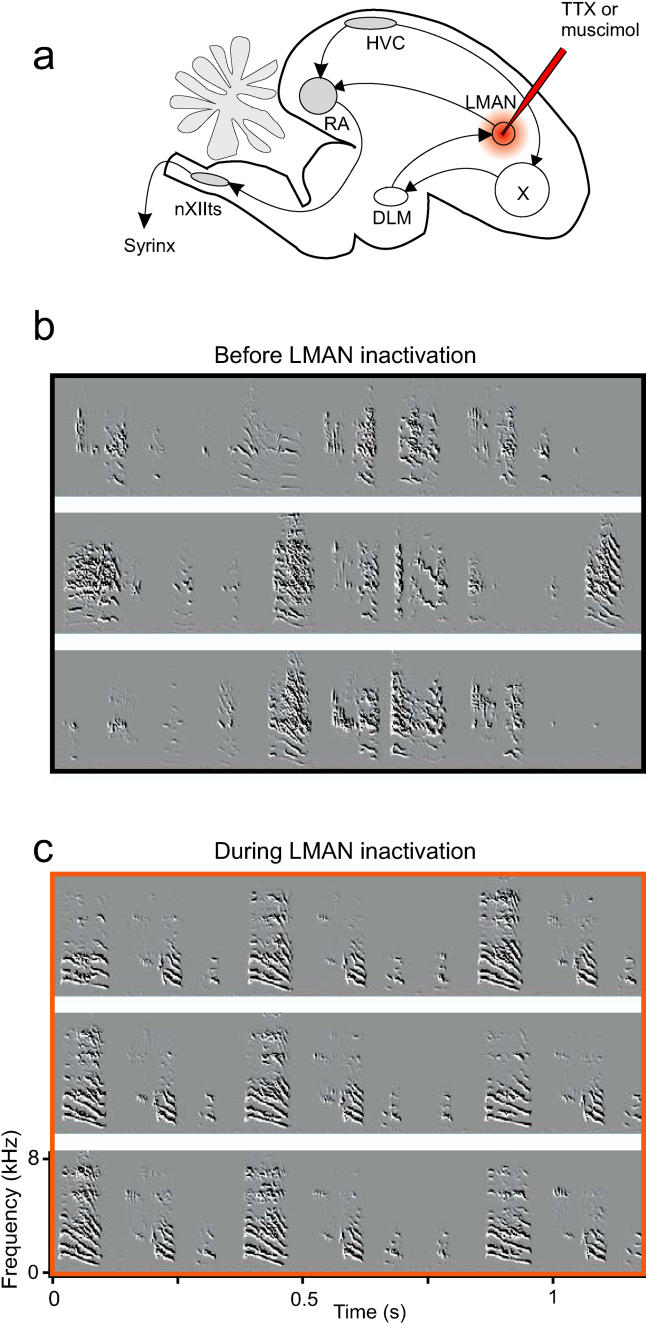
Inactivation of LMAN Significantly Reduces Vocal Experimentation, Making the Otherwise Variable Song of the Juvenile Zebra Finch Highly Stereotyped (A) Two major pathways in the vocal control system of the songbird. The motor pathway (gray) includes motor cortex analogs HVC and RA, while the AFP (white), a basal ganglia thalamo-cortical circuit, consists of Area X, the dorsolateral anterior thalamic nucleus (DLM), and LMAN, which, in turn, projects to RA. To inactivate the output of the AFP, injections of TTX and muscimol (red bolus) were made into LMAN. (B) Examples of a juvenile zebra finch song (57 dph) showing large variability in the sequence and acoustic structure of song syllables. (C) Inactivating LMAN with TTX produces an immediate reduction of sequence and acoustic variability, revealing a highly stereotyped song produced by the motor pathway. The song snippets shown in (B) and (C) are from consecutive song bouts, immediately before and 1 h after drug injection. Songs are displayed as spectral derivatives calculated as described [36]. The frequency range displayed is 0–8.6 kHz. For audio of song bouts before and during LMAN inactivation in this bird, refer to Audios S1 and S2, and S3 and S4, respectively.

In the songbird, two main neural pathways are involved in song production and song learning (Figure 1A). The “motor pathway” controls the vocal motor program through the hierarchical organization of several premotor nuclei [4]. A key nucleus in the motor pathway is the robust nucleus of the arcopallium (RA), which projects to brainstem nuclei controlling the vocal and respiratory muscles [5]. During singing, RA neurons in adult birds generate a highly stereotyped sequence of bursts [6,7], which appear to be driven by precisely timed inputs from a higher premotor vocal area, nucleus HVC [8]. RA also receives input from the “anterior forebrain pathway” (AFP), a circuit homologous to the basal ganglia thalamo-cortical loops [9,10] that may be involved in controlling motor behavior and stereotypy in mammals [11]. Lesions of the AFP in juvenile zebra finches have devastating effects on song development, whereas the same manipulations in adults have few short-term consequences for song production [12,13].

While the critical importance of the AFP for song learning has been established, its specific role remains unknown [14]. It has been proposed that the AFP may be involved in comparing the auditory feedback of the bird's vocal output with a stored auditory template of the desired song—an evaluation process that could provide a corrective signal to the motor pathway needed for learning [15]. However, recent results showing that the firing patterns of neurons in the lateral magnocellular nucleus of the nidopallium (LMAN) of adult birds are insensitive to distorted auditory feedback have called this idea into question [16,17]. Here we test the alternative hypothesis that, in juvenile songbirds, LMAN is involved in generating vocal variability [18]—the other important ingredient of reinforcement learning.

## Results

Our approach was to transiently inactivate LMAN in juvenile zebra finches (*n* = 7 birds, see Materials and Methods), and observe whether and how their songs were affected. Birds were briefly head-restrained, and injections of a sodium channel blocker, tetrodotoxin (TTX, 30 nl, 50 μM), were made in LMAN in both hemispheres, inactivating the nucleus (see Figures S1 and S2). After injections, birds were returned to a sound-isolated chamber, where they typically began to sing after 0.5–1.5 h. In all birds probed, LMAN inactivation resulted in an immediate loss of acoustic variability across song renditions. The effect was particularly dramatic in birds at an early stage of song development (approximately 55 d post hatch [dph]) because these birds normally exhibit greater song variability (Figures 1B, 1C, and S3; Audios S1–S4).

**Figure 2:**
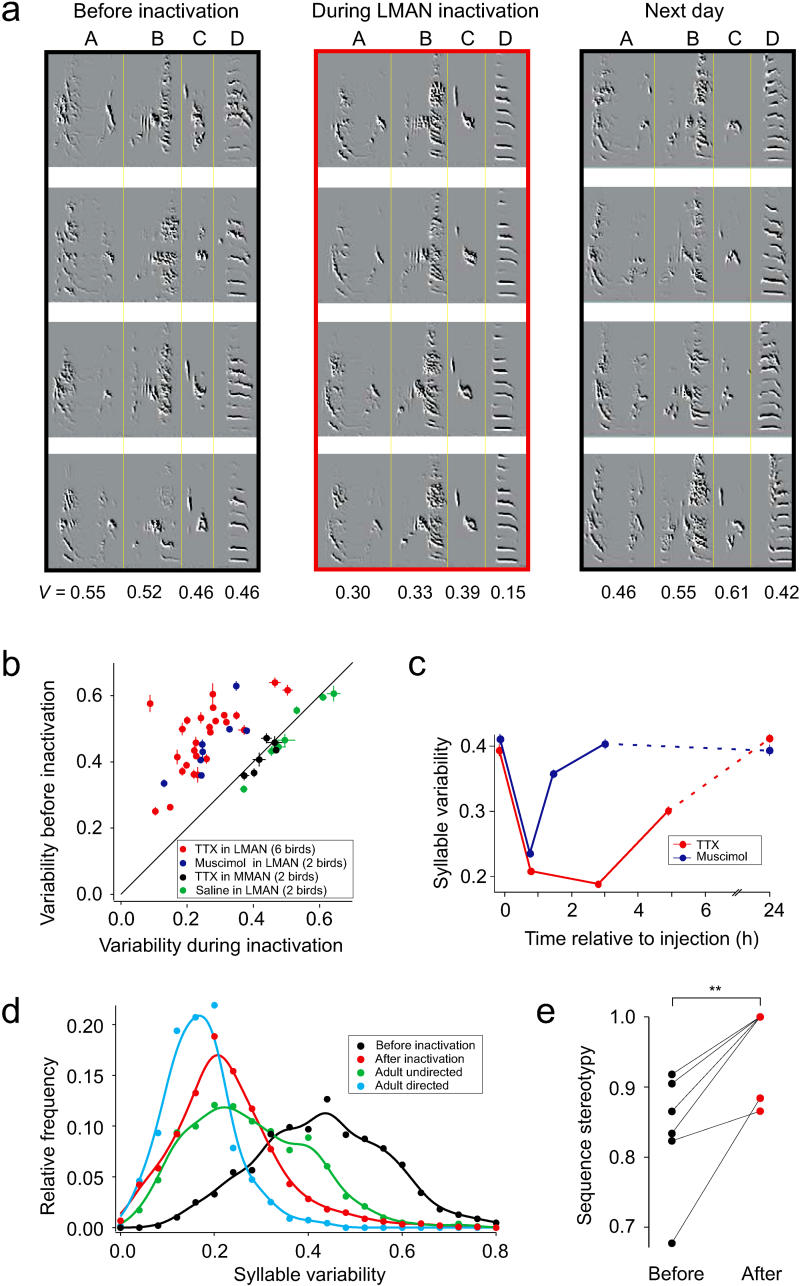
Analysis of the Effect of Bilateral LMAN Inactivation on Song Variability (A) Consecutive renditions of a repeating song motif of 0.5 s duration in a juvenile bird (59 dph) arranged vertically. Note the large variations in acoustic structure within individual syllables before LMAN inactivation (left). Following TTX injection into LMAN, the acoustic variability is dramatically reduced (middle), only to return to the original level by the following day (right). Numbers below each column indicate the variability index (See Materials and Methods section) calculated for the four renditions of the syllables shown. (B) Scatter plot of variability scores before and during LMAN inactivation with TTX (red) and muscimol (blue). Also shown are results for bilateral TTX injection into MMAN (black; see text), and saline injection into LMAN (green). (C) Time course of variability reduction following TTX (red) and muscimol (blue) injections show a time dependence that reflects the known in vivo pharmacology of the respective agents. Data were averaged over four identified syllables and taken from the same bird over consecutive days (dph = 70 and 71; muscimol inactivation followed by TTX inactivation). (D) Distribution of variability scores for all syllables analyzed in the TTX and muscimol experiments (25 unique syllables, six birds) before (black) and during (red) LMAN inactivation in juvenile birds. Shown for comparison are the variability scores for adult zebra finch syllables (18 syllables, 4 birds; undirected song, green; directed song, light blue). Dots represent raw data, while the lines are smoothed running averages. (E) TTX inactivation of LMAN significantly increased syllable sequence stereotypy. Sequence stereotypy scores (see Materials and Methods) for six birds before (black) and after (red) TTX injections into LMAN. For comparison, the average stereotypy score for adult birds singing directed song was 0.95 (*n* = 4 birds).

To quantify song variability, experiments were carried out in slightly older birds with less sequence and acoustic variability (*n* = 6 birds; age range, 59–72 dph) (Figure 2). This allowed us to reliably identify song syllables, the basic acoustic units of zebra finch song, across song renditions (Figure 2A). The variability score *(V)—*a measure reflecting the acoustic variability of a syllable across renditions (see Materials and Methods)—was calculated for all identified syllables before and after TTX injection. Without exception, the syllables showed a highly significant reduction in variability as a consequence of LMAN inactivation (Figure 2B; *n* = 25 syllables; 〈*V*〉_before_ = 0.46, 〈Δ*V*〉 = 0.2; *p*_ave_ < 0.0001, *t*-test). In fact, the juvenile song after inactivation was significantly less variable than songs of adult zebra finches singing undirected song (i.e., songs not directed to a female; Figure 2D; *p* < 0.001, *t*-test). LMAN inactivation also eliminated 75% of the difference in mean variability between juvenile song and adult directed song—the most highly stereotyped form of song [19].

To verify that the loss of variability resulted from silencing LMAN neurons, and not from inactivating fibers of passage near LMAN, a GABA_A_ receptor agonist (muscimol, 30 nl, 25 mM) was injected bilaterally into LMAN (*n* = 2 birds; 66 and 70 dph). Again, all syllables showed a dramatic reduction in variability after injection (*n* = 8 syllables; 〈*V*〉_before_ = 0.43, 〈Δ*V*〉 = 0.16; *p*_ave_ < 0.0001, *t*-test). While the reduction in acoustic variability was similar to that resulting from TTX injections (Figure 2B), the duration of the effect of muscimol was substantially shorter than observed for TTX (Figure 2C). This difference in temporal profile was in good agreement with the known in vivo pharmacology of TTX and muscimol [20,21], suggesting a direct link between suppression of spiking activity in LMAN and loss of song variability.

An additional effect of LMAN inactivation was a significant reduction in sequence variability, a measure of the variability in syllable ordering (Figure 2E; *p* < 0.005, paired *t*-test; see Materials and Methods). In fact, the sequential ordering of syllables after TTX injection was comparable in stereotypy to that of adult song. Thus, LMAN activity may influence sequence generation, possibly through an indirect feedback pathway going from RA to HVC, the putative sequence generator [6,8,22].

We confirmed that the loss of song variability following injections into LMAN did not result from diffusion of the drugs into the medial magnocellular nucleus of the nidopallium (MMAN), a nucleus approximately 1.25 mm medial from LMAN with projections to HVC. Bilateral injections of TTX into MMAN, done in the same birds in which LMAN injections were previously made, had no significant effect on acoustic variability (Figure 2B).

**Figure 3:**
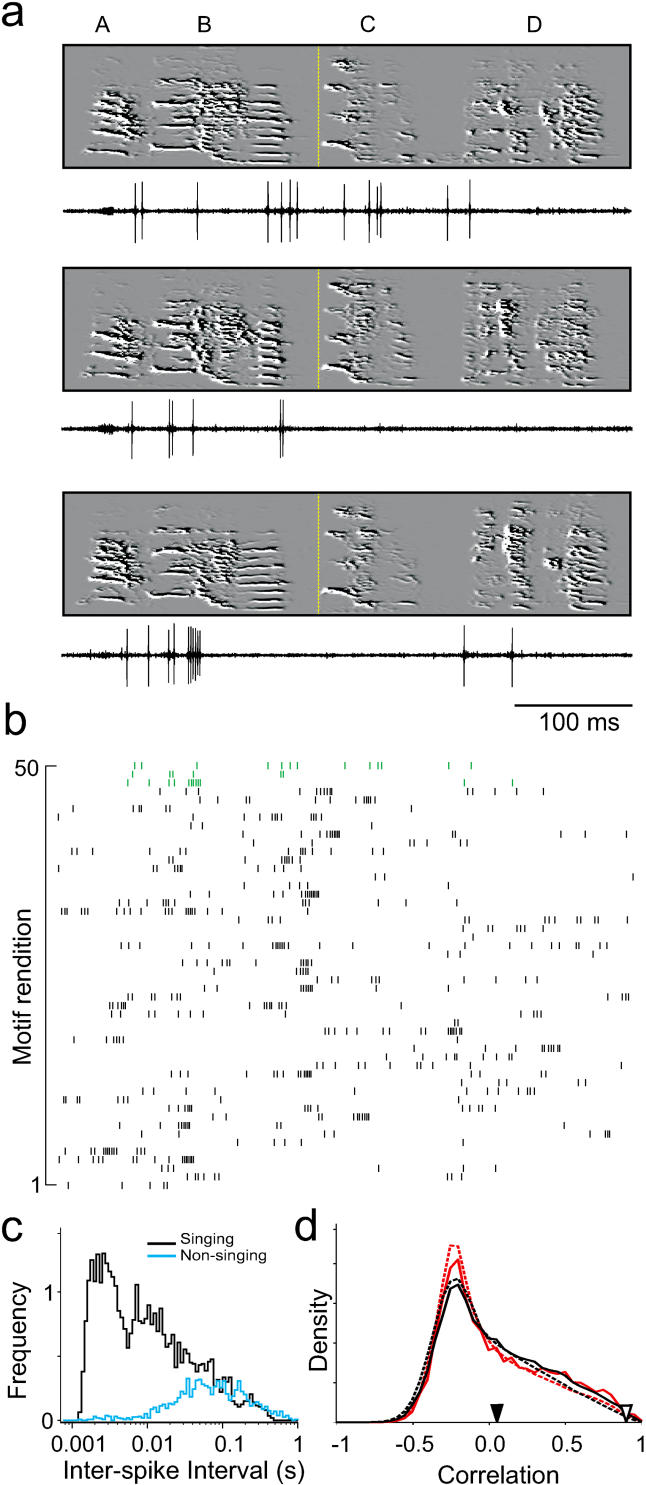
Song-Aligned Firing Patterns of RA-Projecting LMAN Neurons in Singing Juvenile Zebra Finches Are Highly Variable (A) Three successive renditions of a 67-d-old bird's song motif. Displayed under each spectrogram is the simultaneously recorded voltage waveform of an antidromically identified RA-projecting LMAN neuron (verified by collision testing). Average syllable variability for the three motifs is 0.31. Motif alignment was done at the onset (yellow lines) of syllable C. (B) Raster plot showing the spike patterns for 50 consecutive motif renditions for the same cell as in (A). The motifs from (A) are indicated in green. (C) Relative frequency of inter-spike intervals during singing (black) and non-singing (blue) for all the 17 identified projection neurons (units are intervals per second; bin size is 0.04 log units). (D) Distribution of spike-train correlations across all pairs of motifs for the cell in (B) (solid red line). Correlations calculated with random time shifts added to the spike trains have a similar distribution (dashed red line; see Materials and Methods). Also shown is the correlation distribution for the population of identified projection neurons (solid black line; mean correlation indicated by solid arrowhead), and for the population with random time shifts added (dashed black line). In comparison, spike trains of neurons in premotor nucleus RA of the adult bird are highly stereotyped (from [23]; mean correlation indicated by open arrowhead).

We next considered the neural mechanisms by which LMAN affects variability in the motor pathway. One intriguing possibility is that song variability is driven by fast synaptic input from LMAN. If true, then acoustic variability should be accompanied by variability in the firing patterns of RA-projecting LMAN neurons. To test this idea explicitly, we recorded single-unit signals from 29 LMAN neurons in singing juvenile birds (*n* = 3 birds; age range, 62–79 dph) (Figure 3). In all, 17 of these were antidromically identified as RA-projecting LMAN neurons (see Materials and Methods). These neurons exhibited song-related changes in firing rate (spontaneous activity, 12 ± 4 Hz; during singing, 39 ± 6 Hz [mean ± standard deviation]), and generated significantly more bursts during singing (Figure 3C). Raster plots of the spike trains aligned to the song motif showed that the patterns of spikes and bursts generated by individual neurons were different each time the bird sang (Figure 3A and 3B).

Correlations in the spike trains across different renditions of the motif were small (0.054 ± 0.34 [mean ± standard deviation]) compared to those observed in premotor neurons of adult birds (0.90 ± 0.1) [7]. We also compared the correlation distributions to those calculated after random time shifts were added to the spike trains (see Materials and Methods). In general, the correlation distributions of the randomized spike trains were very similar to those calculated for the motif-aligned spike trains (Figure 3D), confirming that the firing patterns of LMAN neurons are highly variable. Nevertheless, in 13 out of the 17 identified RA-projecting neurons the correlation distributions were still significantly different from those of the randomly shuffled spike trains (*p* < 0.01, Kolmogorov-Smirnov test), suggesting that while LMAN activity is highly variable, it is not completely random with respect to the song.

**Figure 4:**
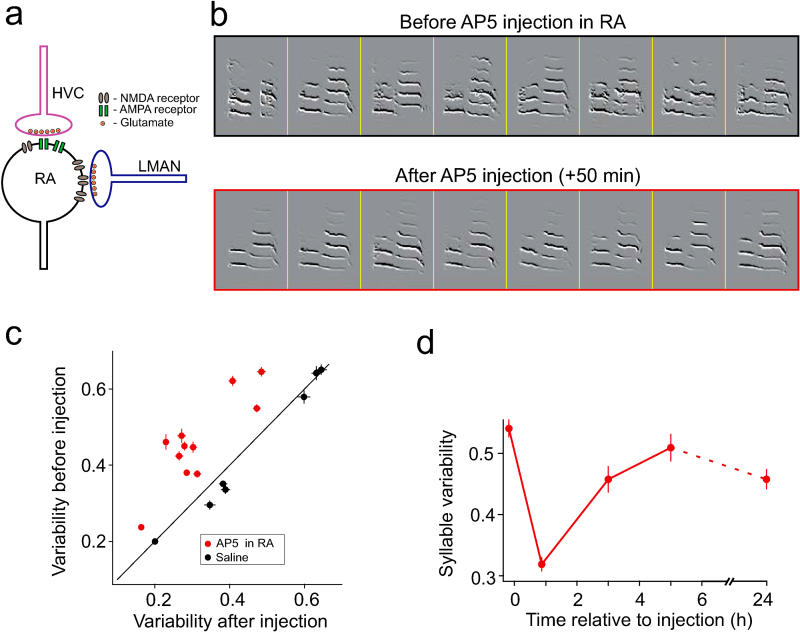
Bilateral Injections of the NMDA Receptor Antagonist AP5 into RA Significantly Reduced Song Variability (A) Excitatory synaptic inputs to RA from LMAN and HVC are mediated by a different mix of glutamate receptor types (see text). Using AP5 we could block LMAN input while only partially inactivating HVC input. (B) Eight sequential renditions of one song syllable in a juvenile zebra finch (63 dph) before and after AP5 injection into RA. Note the rapid fluctuations in pitch, the appearance of noisy acoustic structure, and variations in syllable duration before injection. The average variability scores *(V)* before and after injections for the eight shown syllable renditions were 0.50 and 0.25, respectively. (C) Following injection of AP5 into RA, fluctuations in acoustic structure were substantially reduced. Variability scores of 11 syllables in four birds before and after injection of AP5 into RA. (D) Time course of acoustic variability following drug injection averaged over all identifiable syllables for the bird in (B).

Guided by the neural data, we next tested the hypothesis that LMAN drives song variability by providing excitatory glutamatergic input to RA—which in the zebra finch is mediated almost exclusively by N-methyl-D-aspartate (NMDA)–type receptors [24]. In contrast, glutamatergic inputs to RA from HVC are mediated by a mixture of NMDA and α-amino-3-hydroxy-5-methyl-4-isoxazolepropionic acid (AMPA)–type receptors (Figure 4A) [25]. Thus, if LMAN drives song variability through glutamatergic input to RA, then blocking NMDA receptors should reduce this variability, while sparing the AMPA-mediated drive from HVC. In line with our hypothesis, bilateral injections of the NMDA receptor antagonist 2-amino-5-phosphonovalerate (AP5, 50 nl, 30 mM) into RA significantly reduced acoustic variability in all song syllables examined (Figure 4B and 4C; *n* = 4 birds; age range, 57–73 dph; 11 syllables; 〈*V*〉_before_ = 0.47, 〈Δ*V*〉 = 0.16; *p*_ave_ < 0.0001, *t*-test). The time course of the variability reduction (Figure 4D) was consistent with the temporal profile of AP5 effects seen in other in vivo studies [26].

Given that AP5 has effects beyond blocking LMAN input to RA, it may influence the song in ways other than reducing variability. To examine whether AP5 injections affected the acoustic structure of syllables, we compared the acoustic features of syllables after AP5 injection to the same syllables before injection (average similarity score 78.0, 11 syllables; see Materials and Methods). In comparison, the average similarity score across renditions of the same syllables prior to injection was 77.7, suggesting that the effect of AP5 injection was largely limited to song variability.

## Discussion

Previous studies have shown that permanent LMAN lesions in the juvenile bird disrupt song learning and result in an impoverished and prematurely stereotyped song [12,13]. Such lesions are known to produce synaptic maturation in RA within a few days [27], perhaps because of a loss of neurotrophic input from LMAN [12,13]. Because of the long delay from lesioning to singing (often several days), these studies could not address whether increased stereotypy was caused by synaptic reorganization in RA, or by a more immediate mechanism such as the loss of fast synaptic input from LMAN. In our experiments, we observe singing within an hour after injection, and find that LMAN inactivation reduces song variability reversibly and on a short timescale. This observation implies that, in addition to slow neurotrophic effects, LMAN acts on RA rapidly to drive or control song variability, a necessary ingredient of reinforcement learning. Thus, our results suggest that the loss of vocal plasticity following permanent lesions of LMAN may, in part at least, be due to the immediate loss of exploratory behavior.

What is the mechanism by which neural activity in LMAN controls motif-to-motif variability in the song? Our experiments tested the hypothesis that fluctuations in the song are driven directly by synaptic input from LMAN [25]. In this view, the premotor circuit generates a stereotyped song sequence upon which the AFP acts to drive variations. This hypothesis requires that neural activity in LMAN be highly variable across different song motifs, a prediction that was borne out by our recordings in LMAN (see Figure 3). In comparison, premotor neurons in adult birds (singing song of comparable stereotypy to our LMAN-inactivated juvenile birds) generate extremely stereotyped, song-locked spike patterns [6,7, 8]. In itself, the result that LMAN neurons are only weakly time-locked to the song may not be surprising. The significance of this observation becomes apparent when considering that these neurons send excitatory projections to the motor pathway, and that they are necessary for the expression of song variability as demonstrated by our inactivation results. Together with the finding that electrical stimulation of LMAN in adult birds can drive transient changes in the song [19], these observations make LMAN a likely source for the variability in the premotor pathway.

Because LMAN input to RA neurons is mediated almost exclusively by NMDA receptors, another strong prediction of our hypothesis was that blockade of NMDA receptors in RA should reduce song variability. Our results from the injection of AP5 into RA confirmed this. However, given the presence of NMDA receptors in the projection from HVC to RA [24], and perhaps in recurrent connections within RA, blockade of NMDA receptors is likely to have effects on RA circuitry beyond the loss of direct synaptic input from LMAN. Thus, this experiment cannot preclude other hypotheses—for example, that LMAN acts to regulate stochastic processes intrinsic to the premotor circuit, through some yet unknown mechanism.

Further support for the idea that LMAN can drive song variations comes from studies in the adult zebra finch. Song-related neural activity in LMAN is variable also in the adult bird, and this variability has been shown to be larger during undirected as compared to directed singing [27,28]. A recent study [19] linked the increased neural variability in LMAN during undirected singing to an increase in motif-to-motif variability in song features (see also Figure 2D).

How does the role and function of LMAN change as song variability is reduced during learning and finally during song crystallization? To the extent that the variability of LMAN firing patterns in the adult bird during undirected song [28] is similar to that in the juvenile bird, an essential part of song development may be a reduction of the gain by which LMAN drives RA. This could occur as a result of synaptic changes within RA that weaken input from LMAN and/or strengthen the projections from HVC. While there is evidence that this may indeed occur [26,29], more experiments are needed to establish how the developmental reduction in song variability is related to changes in song circuitry.

Reinforcement learning requires that variability in the motor output be accompanied by a mechanism that evaluates the resulting performance. In the songbird, such an evaluation signal could be sent directly to the motor system (e.g., to RA), perhaps via a neuromodulator [30, 31], to reinforce the states of the motor pathway that lead to a better-than-expected match to the memorized template. A reinforcement signal could also be sent to the AFP to shape or regulate the fluctuations introduced into the motor pathway via LMAN. This would make LMAN more than a simple “noise generator,” allowing it to bias vocal fluctuations in the direction of the desired song. Such bias is suggested by the presence of small but significant correlations in the motif-aligned firing pattern of LMAN neurons (see Figure 3). This bias could permit a more efficient exploration of motor space, and even allow LMAN activity to drive plastic changes in the motor circuitry.

The exploratory motor behavior exhibited by juvenile songbirds may also provide general insights into how the brain generates fluctuations required for learning. Such fluctuations could be generated within the motor pathway or by brain regions projecting to it, and could result from stochastic processes, such as randomness in synaptic release [32], noise propagated by summation of irregular patterns of inhibitory postsynaptic potentials and excitatory postsynaptic potentials [33], or complex collective dynamics of the neuronal network [34]. Our results strongly suggest that, whatever the detailed biophysical mechanisms, the neural circuits generating these fluctuations are located outside the motor pathway in a specialized pathway involving the basal ganglia. The output of this circuit acts on the motor pathway, allowing the song system to explore the vocal space in a purposeful manner. Whether inducing exploratory motor behavior is a general feature of basal ganglia circuits is an intriguing idea that remains to be explored.

## Materials and Methods

### 

#### Subjects

Subjects were juvenile male zebra finches (54–79 dph). Birds were obtained from the Massachusetts Institute of Technology zebra finch breeding facility (Cambridge, Massachusetts), and from the aviary at the Rockefeller Field Research Station (Millbrook, New York). The care and experimental manipulation of the animals were carried out in accordance with guidelines of the National Institutes of Health and were reviewed and approved by the Massachusetts Institute of Technology Institutional Animal Care and Use Committee.

#### Reversible inactivation

Birds underwent a brief surgery to attach to the skull a means of restraining the head during drug injections. The animals were anesthetized with isoflurane (2%) and placed in a stereotaxic apparatus (MyNeuroLab.com, St. Louis, Missouri, United States). Two stainless-steel screws (#0–80 6 mm long) were secured to the skull with dental acrylic. Small holes (approximately 300 μm in diameter) were drilled through the cranium bilaterally over LMAN or MMAN, or RA using stereotaxic coordinates. The holes were covered with a thin layer of Kwik-Kast (World Precision Instruments, Sarasota, Florida, United States). The animals were then placed in a custom sound-isolation chamber where they began to sing prolifically after a few days—typically 200–1,000 song motifs per hour.

Inactivation of song control nuclei in the singing bird was carried out by placing the bird, unanesthetized, in a small foam restraint and attaching the head-mounted screws to a metal plate bolted to the stereotaxic apparatus. The Kwik-Kast over the cranial holes was removed, and 30 nl of TTX (50 μM, #T5651, Sigma, St. Louis, Missouri, United States) or muscimol (25 mM, #M1523, Sigma) was injected bilaterally into the brain region of interest using a Nanoject II injector (Drummond Scientific, Broomall, Pennsylvania, United States). The procedure of injecting the birds took approximately 10 min. Experimental confirmation of the physiological effects of TTX injections showed that LMAN was likely completely inactivated after our injections (see Figure S2). Regions immediately surrounding LMAN were also affected, and we cannot rule out an indirect contribution from the partial inactivation of these regions. For inactivation of NMDA-mediated synapses in RA, AP5 (#A5282, Sigma) was injected bilaterally into RA (50 nl, 30 mM). The injection site was guided by electrophysiological recordings of spontaneous activity in RA.

Injected solutions also contained dye-conjugated dextrans (#D22912, Molecular Probes, Eugene, Oregon, United States). All injection sites were verified by histological examination and were found to be within the target nucleus (see Figure S1), except for TTX injections in LMAN in two birds: one in which the LMAN injection site in one hemisphere was found to be approximately 100 μm anterior to the edge of LMAN, the other in which the injections were approximately 200 μm posterior to LMAN, but right in the middle of the fiber tract leading from LMAN to RA. The results from these birds were similar to those from other birds, and were included in the analysis.

#### Chronic neural recordings in LMAN

Experiments were timed such that the birds were at an age at which they produced readily identifiable syllable sequences, yet showed variable acoustic syllable structure across song renditions. Recordings were carried out using a motorized microdrive described previously [35]. Cells were isolated by searching for spontaneous or antidromically evoked spiking activity; units typically had signal-to-noise ratios greater than 10:1. Antidromic identification of RA-projecting LMAN neurons was carried out with a bipolar stimulating electrode implanted in RA using techniques described previously for antidromic identification of RA-projecting HVC neurons [8]. Neurons exhibiting a short-latency antidromic spike (<5 ms) with a root-mean-squared latency jitter of less than 100 μs (at a stimulation current of approximately 10% above threshold) were counted as identified RA-projecting neurons. Of the 17 antidromically identified neurons in our dataset, ten were further validated with collision tests [8]. An additional ten putative projection neurons did not respond to RA stimulation with a short-latency spike, but exhibited spike patterns and correlations similar to the identified projection neurons. For the cells in our dataset, we recorded signals for many song motifs (range, 5–133 motifs; mean, 56).

#### Data analysis

To assess the effects of drug injections on acoustic variability and average acoustic structure, analysis was done on reliably identifiable song syllables (range, 2–5 per bird; see Figure 2A for an example). Each data point was derived from 45 pairwise comparisons made across ten consecutive renditions of a given syllable, recorded immediately before and after injection. Acoustic variability was quantified using the Sound Analysis Pro 1.04 software [36], and pairwise comparisons of the acoustic features of identified syllables were made using the local similarity measure (“accuracy”). This measure is based on pitch, frequency modulation, amplitude modulation, Wiener entropy, and goodness of pitch, and is calculated in 9-ms intervals and averaged over the duration of the syllable; syllables were aligned in time so as to maximize the similarity, allowing for 5% time warping. For the variability measurements, the resulting similarity score (S, ranging from zero to 100) was converted, through a linear remapping, to a variability score *(V)* by the following formula:



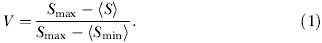



〈*S*_min_〉 is the average similarity score of randomly chosen pairs of syllables from unrelated birds, which in our finch colony was measured to be 50 ± 12 (mean ± standard deviation, *n* = 200 pairwise comparisons; comparisons were made across syllables of birds from different fathers). The similarity of identical syllables, *S*_max_, is 100 by definition of the similarity measure. Thus, a variability score of one means that syllables are as different as two unrelated syllables would be on average, while variability score of zero means that the syllables are identical. Error bars for *V* in the figures all denote standard error of the mean. 〈*V*〉 denotes the average variability score across birds and syllables for a given condition.

The variability of syllable ordering in a song was quantified using the stereotypy score of Scharff and Nottebohm [13], excluding the variability in the number of introductory notes and in the end syllable of a song bout. The score is a combination of “sequence linearity,” which addresses the way in which notes are ordered, and “sequence consistency,” a measure of the frequency with which the main motif sequence appears. Complete stereotypy yields a score of one, while a completely random sequencing will have a score close to zero. Stereotypy scores were calculated over ten consecutive song bouts, before and after LMAN injections.

For the analysis of the neural recordings in LMAN, we determined the sequence of song syllables most frequently produced by each bird. Motifs that matched this sequence were identified and time-aligned using the onset of one of the syllables. The alignment syllable was chosen for a sharp onset in acoustic power. The relative jitter in the timing of other syllables in the motif was found to be less than 9 ms (root mean squared). Spike times were extracted, and the instantaneous firing rate during each motif rendition was estimated by smoothing the spike train with a Gaussian of half-width 20 ms (to the 1/e points). Correlations were calculated between the firing rate functions for all pairs of smoothed spike trains. Correlations were also calculated for all pairs of spike trains after a random time shift. The shift was circular, such that spikes wrapped around to the beginning of the motif; time shifts were chosen randomly from a uniform distribution with the width of the motif. For each cell the correlation distribution of the time-shifted firing rates was calculated with 100 different ensembles of random shifts. This random shift ensured zero mean correlation while preserving spike statistics. Thus, the distribution of time-shifted correlations provides a zero-correlation baseline with which to compare our results.

## Supporting Information

Figure S1 Histology Confirming the Injection Sites for the LMAN Inactivation Experiments in Figures 1 and 2
(A) A parasaggital Nissl-stained section of a zebra finch brain showing the location of LMAN.
(B) Inverted darkfield image showing LMAN in one of the juveniles injected (red markers in [D] and [E]).
(C) Combined darkfield and fluorescence image showing the spread of the dye that was co-injected with the drug.
(D and E) Estimated injection sites relative to the boundaries of LMAN for all birds in Figures 1 and 2 in the saggital (D) and coronal (E) planes, respectively (individual birds are color coded).
(F) Estimated maximum diameter of LMAN in the saggital plane.
(G) Estimated lateral extent of LMAN in the coronal plane.
The estimates in (F) and (G) are based on the contrast borders seen in the darkfield images (see [B]). Note that fibers from LMAN to RA leave the posterior edge of LMAN.
(369 KB PDF).

Figure S2 Dose- and Distance-Dependent Effects of TTX Injections in and around LMAN (A) Decrease in acoustic variability (ΔV) approximately 1 h after injection, as a function of location and concentration of TTX injections. Red bars indicate dose response for TTX injections in LMAN (*n* = 2 birds; 8 syllables; injection sites for the two birds correspond to the blue and grey markers in Figure S1). Blue bars indicate 30-nl saline injections in LMAN (*n* = 2 birds; 7 syllables). Green bars indicate 30-nl (50 μM) TTX injections 1.25 mm medial (MMAN, *n* = 2 birds; 6 syllables) and dorsal (“above,” *n* = 2; 8 syllables) from the center of LMAN.
(B and C) Summary of experiments done to verify the physiological spread of TTX. Experiments were done in anesthetized birds (2% isoflurane). A bipolar stimulating electrode was placed in RA, and a recording electrode in LMAN, producing antidromically evoked activity in LMAN (stimulus pulses, 175 μA, 0.2 ms, 0.5 Hz ). TTX (30 nl, 50 μM) was injected at different distances away from the recording electrode. (B) Examples of recorded signals for TTX injections 400 μm (top) and 1,250 μm (bottom) away from the recording electrode (averaged over 30 stimulus pulses). The baseline stimulus artifact recorded 1 mm above LMAN is shown in the green boxes (left). Signal recorded in LMAN immediately before injection is shown in the black boxes (middle). Signal recorded 1 h after injection is shown in the red boxes (right). (C) Summary of evoked activity 1 h after TTX injections made at different distances away from the recording site. Evoked activity was measured as the root-mean-squared deviation of the signal from the baseline in the interval 1.5–4.5 ms after the stimulation pulse (six birds, two at 400 μm, two at 600 μm, and one each at 800 μm and 1,250 μm).
(1.1 MB PDF).

Figure S3 Example of a Juvenile Zebra Finch Song (54 dph) Showing a Loss of Sequence and Acoustic Variability following LMAN Inactivation by TTX Injection The song snippets shown are from three consecutive song bouts, immediately before and 1 h after TTX injection. Tutor song is shown for comparison.
(1.8 MB PDF).

Audio S1 Example of a Song from the Bird in Figure 1 prior to TTX Inactivation of LMAN (Bout 1) (545 KB WAV).

Audio S2 Example of a Song from the Bird in Figure 1 prior to TTX Inactivation of LMAN (Bout 2) (455 KB WAV).

Audio S3 Example of a Song from the Bird in Figure 1 during TTX Inactivation of LMAN (Bout 1) (430 KB WAV).

Audio S4 Example of a Song from the Bird in Figure 1 during TTX Inactivation of LMAN (Bout 2) (360 KB WAV).

## Abbreviations

AFP: anterior forebrain pathway
AMPA: α-amino-3-hydroxy-5-methyl-4-isoxazolepropionic acid
AP5: 2-amino-5-phosphonovalerate
dph: days post hatch
LMAN: lateral magnocellular nucleus of the nidopallium
MMAN: medial magnocellular nucleus of the nidopallium
NMDA: N-methyl-D-aspartate
RA: robust nucleus of the arcopallium
TTX: tetrodotoxin
