# Supplementary material for: Vocal Experimentation in the Juvenile Songbird Requires a Basal Ganglia Circuit
Source: PLoS Biol. 2005 Mar 29;3(5):e153. doi: 10.1371/journal.pbio.0030153 (PMC1069649; doi:10.1371/journal.pbio.0030153)
Supplement: Supplementary file 1 [file pbio.0030153.sg001.pdf]

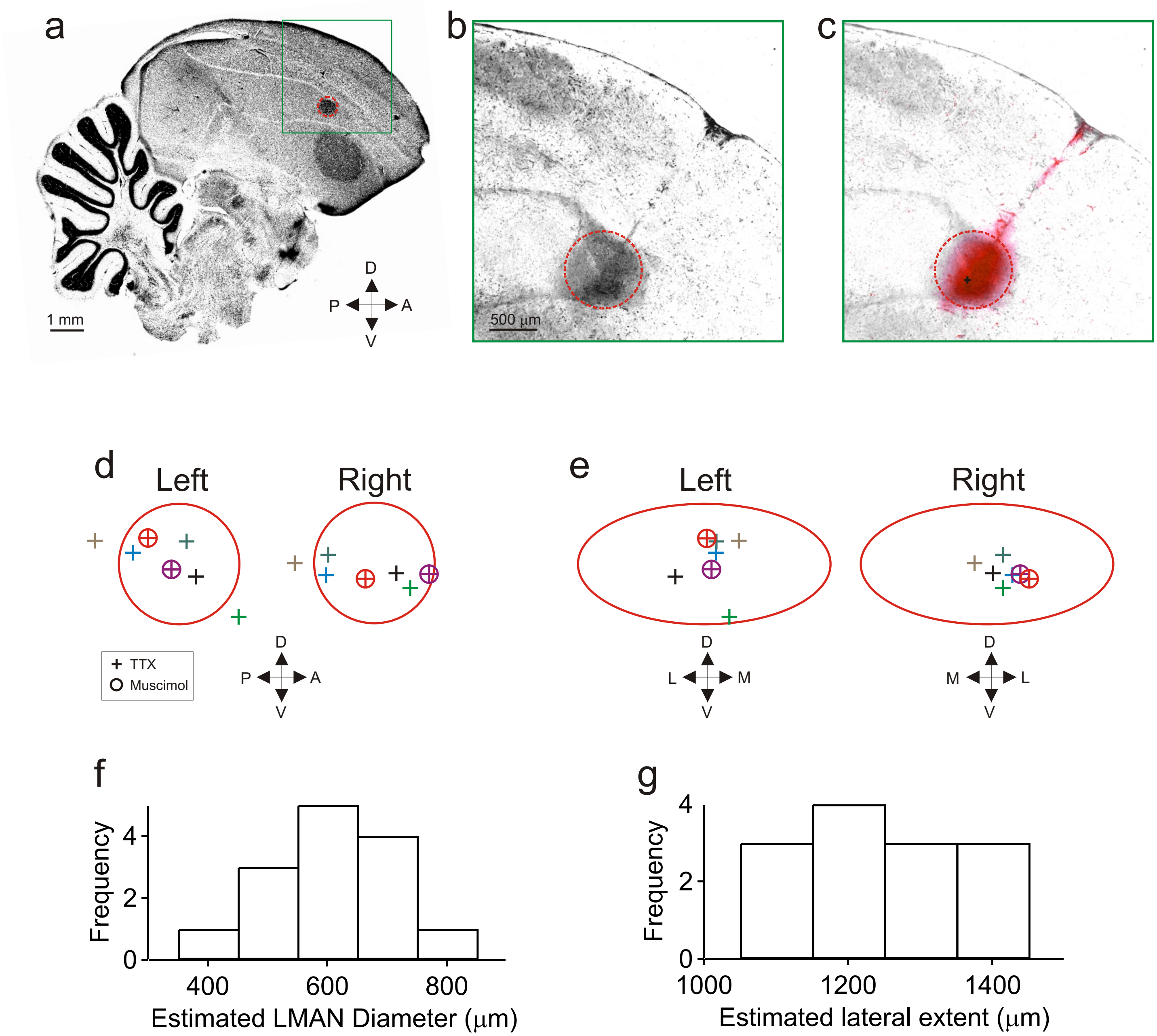

a
b
c
1 mm
500 µm
D
P
A
V
d
Left
Right
TTX
Muscimol
e
Left
Right
L
M
f
Frequency
0
2
4
400
600
800
Estimated LMAN Diameter (µm)
g
Frequency
0
2
4
1000
1200
1400
Estimated lateral extent (µm)
