# Supplementary material for: Vocal Experimentation in the Juvenile Songbird Requires a Basal Ganglia Circuit
Source: PLoS Biol. 2005 Mar 29;3(5):e153. doi: 10.1371/journal.pbio.0030153 (PMC1069649; doi:10.1371/journal.pbio.0030153)
Supplement: Supplementary file 2 [file pbio.0030153.sg002.pdf]

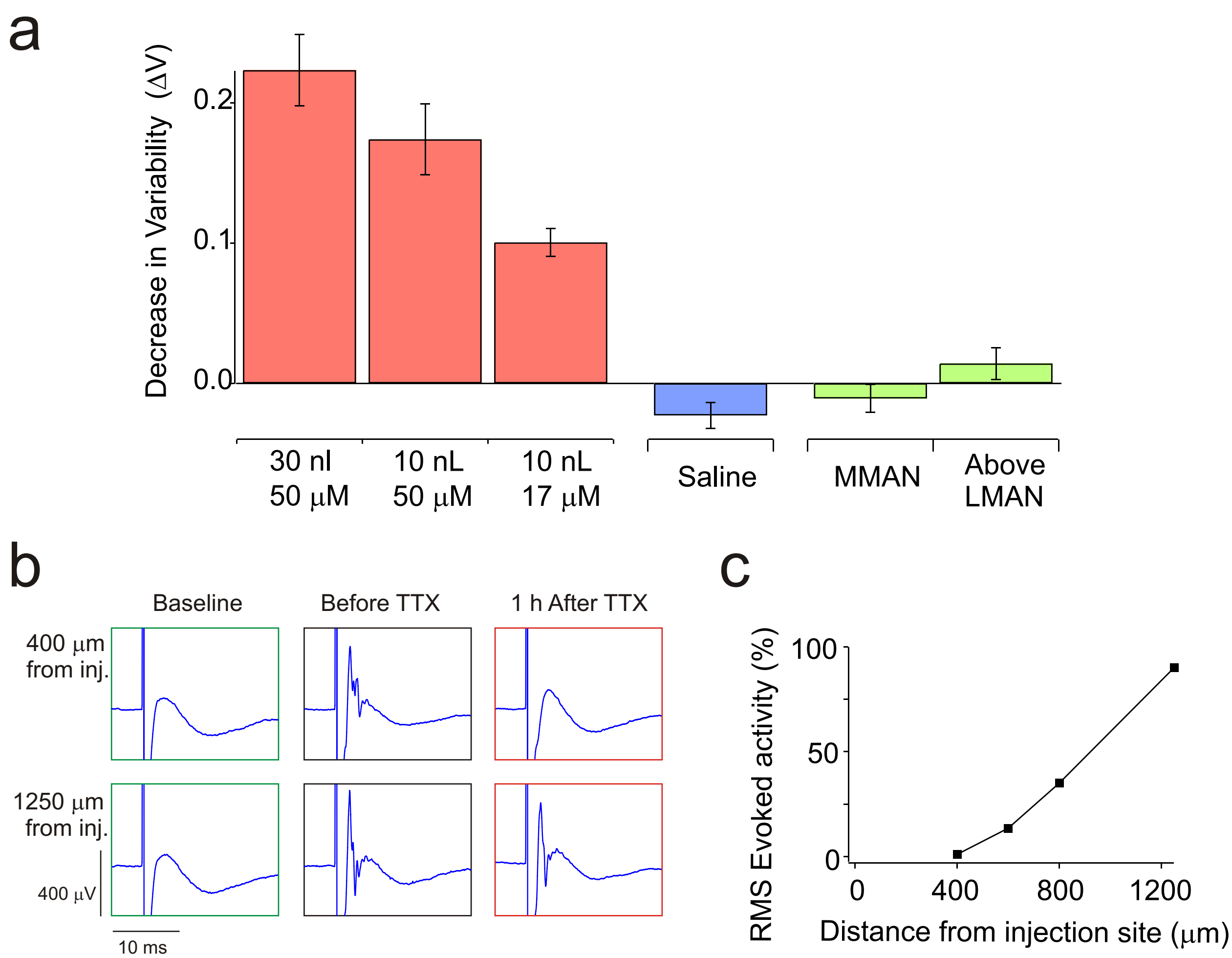
a
Decrease in Variability (ΔV)
0.2
0.1
0.0
30 nl
50 µM
10 nL
50 µM
10 nL
17 µM
Saline
MMAN
Above
LMAN
b
Baseline
Before TTX
1 h After TTX
400 µm
from inj.
1250 µm
from inj.
400 µV
10 ms
c
RMS Evoked activity (%)
100
50
0
0
400
800
1200
Distance from injection site (µm)
