# Supplementary material for: Vocal Experimentation in the Juvenile Songbird Requires a Basal Ganglia Circuit
Source: PLoS Biol. 2005 Mar 29;3(5):e153. doi: 10.1371/journal.pbio.0030153 (PMC1069649; doi:10.1371/journal.pbio.0030153)
Supplement: Supplementary file 3 [file pbio.0030153.sg003.pdf]

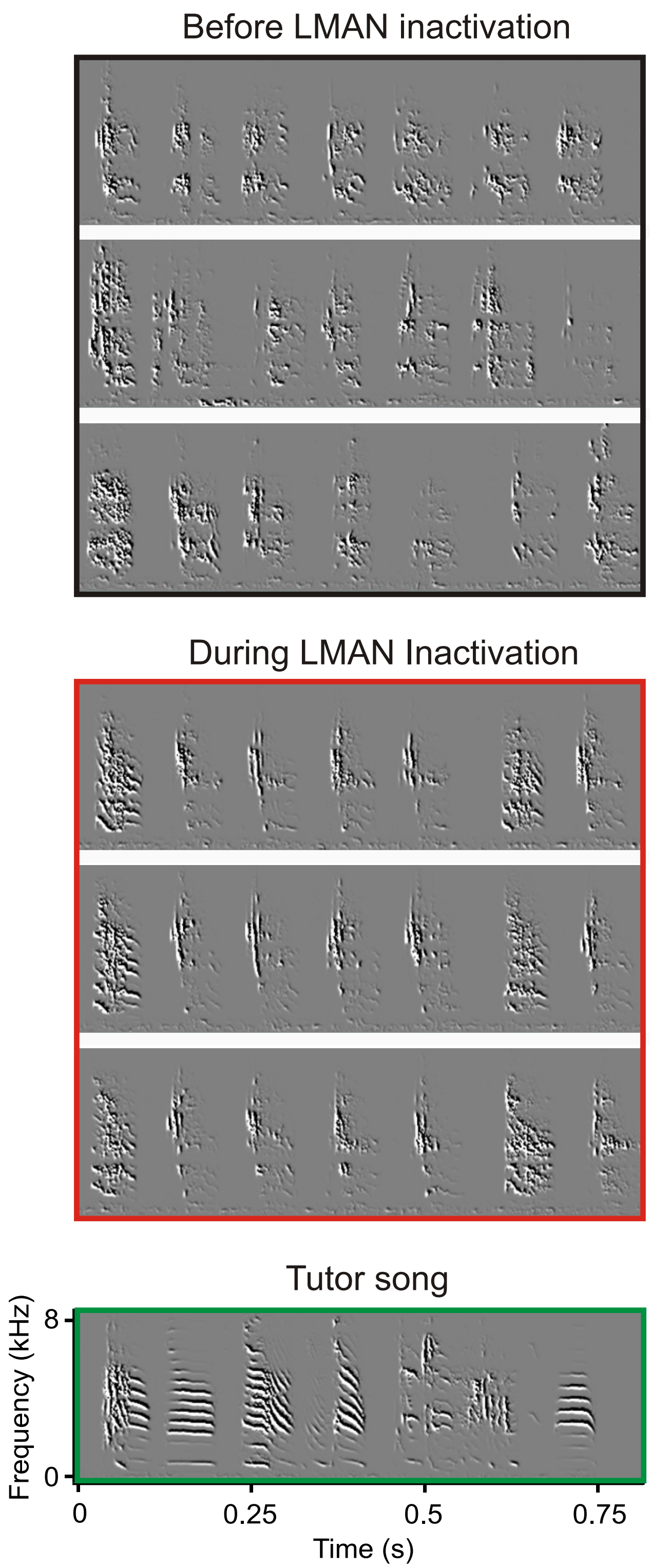
Before LMAN inactivation
During LMAN Inactivation
Tutor song
Frequency (kHz)
8
0
0
0.25
0.5
0.75
Time (s)
